# Is There a Role for Colchicine in the Treatment of Oral Mucous Membrane Pemphigoid?

**DOI:** 10.1111/odi.15193

**Published:** 2024-11-04

**Authors:** E. Fribourg, V. Chuy, M. Fenelon, S. Catros, J. C. Fricain

**Affiliations:** ^1^ Oral Medicine and Chronic Orofacial Pain Unit, Department of Oral Surgery at the Bordeaux University Hospital of Bordeaux Bordeaux France; ^2^ Department of Dentistry and Oral Health University of Bordeaux (CHU Bordeaux) Bordeaux France; ^3^ INSERM and BPH University of Bordeaux Bordeaux France; ^4^ INSERM BioTis, Laboratory for the Bioengineering of Tissues University of Bordeaux Bordeaux France

**Keywords:** colchicine, dapsone, mucous membrane pemphigoid, oral

Mucous membrane pemphigoid (MMP) is the most frequent autoimmune disease encountered in the oral cavity. The main immunological target is the BP‐180 antigen, but the complex mononuclear, granulocytic, and lymphocytic inflammatory reaction results in a cellular and humoral immune response leading to scar fibrosis (Rashid et al. 2021). There is likely a role for treatments targeting innate immunity in mucous membrane pemphigoid. In the absence of randomized controlled clinical trials, there is no established therapeutic consensus supported by scientific evidence for treating oral manifestations of MMP (Schmidt et al. 2021). Based on expert advice, European Guidelines recommend local corticoids as first‐line treatment, and then dapsone in case of failure (Schmidt et al. 2021). Numerous side effects of dapsone have been reported in patients, forcing them to reduce or even discontinue the treatment (Taylor et al. 2015). Furthermore, its efficacy is often limited. The lack of efficacy and poor tolerance of dapsone justify the search for a new treatment. At Bordeaux University Hospital, our team became interested in the potential efficacy of colchicine during a fortuitous therapeutic discovery in 2016. One of our patients, exhibiting severe oral MMP lesions resistant to several therapeutic lines including dapsone (Figure 1), was administered colchicine for rheumatological reasons characterized by ill‐defined distal joint pain. We observed complete remission of MMP oral lesions following the introduction of colchicine (Figure 1). We found another study in the literature which also observed a remission of oral MMP lesions with the use of colchicine on several patients (Chaidemenos et al. 2011). Since this date, we have used colchicine in standard care as a second line of treatment for oral MMP in patients' resistant to topical corticosteroids. The objective of this study was to describe the evolution of patients with oral MMP monitored in our oral medicine department from 2012 to 2021.

**FIGURE 1:**
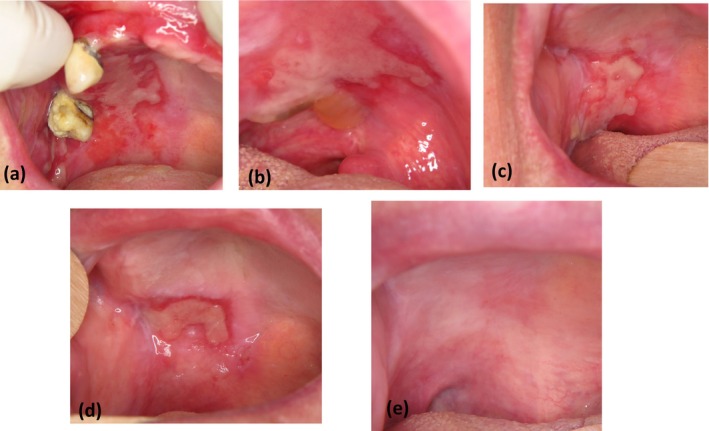
Failure of local corticosteroid, topical tacrolimus, dapsone (a), then of the association of per os corticosteroid therapy with mycophenolate mofetil (b), with azathioprine (c), with local injection of corticosteroid (d), complete remission resolved by colchicine (e).

We conducted a retrospective study on the basis of medical charts of patients admitted for an oral form of MMP, from 2012 to 2021 at the oral medicine department of Bordeaux (University Hospital, France). The study was approved by the Research Ethics Committee of the Center for Ethics and Health Research in Bordeaux: CER‐BDX 2024–193. The three inclusion criteria were as follows: the presence of the “Nikolsky'sign” (Mignogna et al. 2008) and/or post‐bullous ulceration, the presence of a supra‐basal detachment or a blister at the epithelium–chorion junction during histopathological examination, and positive direct immunofluorescence indicating the presence of IgG, C3, IgA, and/or enzyme‐linked immunosorbent assay (ELISA) with targeted identification of anti‐BP‐180 and anti‐BP‐230. The exclusion criteria were as follows: the absence of a follow‐up appointment after first‐line treatment or incomplete medical records for the primary endpoint after first‐line treatment. Remission was considered when the disappearance of “Nikolsky'sign” was reported at each subsequent follow‐up appointment, by the one experimented practitioner in charge of these patients. An informed oral consent was obtained for each patient for the use of self‐care data.

Ninety‐eight eligible patients were identified in the registry of the Department of Oral Medicine. Sixty‐six were not included because they did not meet the three inclusion criteria. Forty‐two patients were included, and 14 patients were excluded because they were lost to follow‐up after the first prescription. Twenty‐eight patients were analyzed median age of 69; sex ratio of 0.3 (6 men–22 women) contributing for a total of 69 prescriptions. The frequency of molecule prescribed is detailed in Figure 2. The first line of treatment used was a topical corticosteroid as clobetasol (41%), with three application/d. In case of failure, we prescribed other molecules in addition to topical corticosteroid, such as per os corticosteroid (17%), colchicine (16%), dapsone (13%), and less frequently mycophenolate mofetil, topical tacrolimus, sulfasalazine, and doxycycline. Oral corticosteroids were prescribed as a short course of 1 mg/kg/d for 1 month, followed by a stepwise decrease of around 2.5 mg to reach 7.5–10 mg/d; colchicine 0.5 mg–1.5 mg/d; and dapsone 50–100 mg/d. At the end of the follow‐up, local corticosteroids alone were effective with the disappearance of “Nikolsky'sign” in 12 out of 23 patients (52.2%), colchicine in 5 out of 6 patients (83.3%), and dapsone in 1 out of 6 patients (16.7%) (Table 1). Six adverse events were experienced with dapsone (anemia, increased methemoglobinemia, hemolysis, pruritus, tremor, tendon pain and muscle weakness, nausea, fatigue, and shortness of breath), resulting in six discontinuations, whereas only two with colchicine (increased transaminases, nausea, leukopenia, neutropenia, and loose stools) resulting in one dosage reduction and one discontinuation. The incidence of adverse events with dapsone was 66.7%, versus colchicine 16.7%.

**FIGURE 2:**
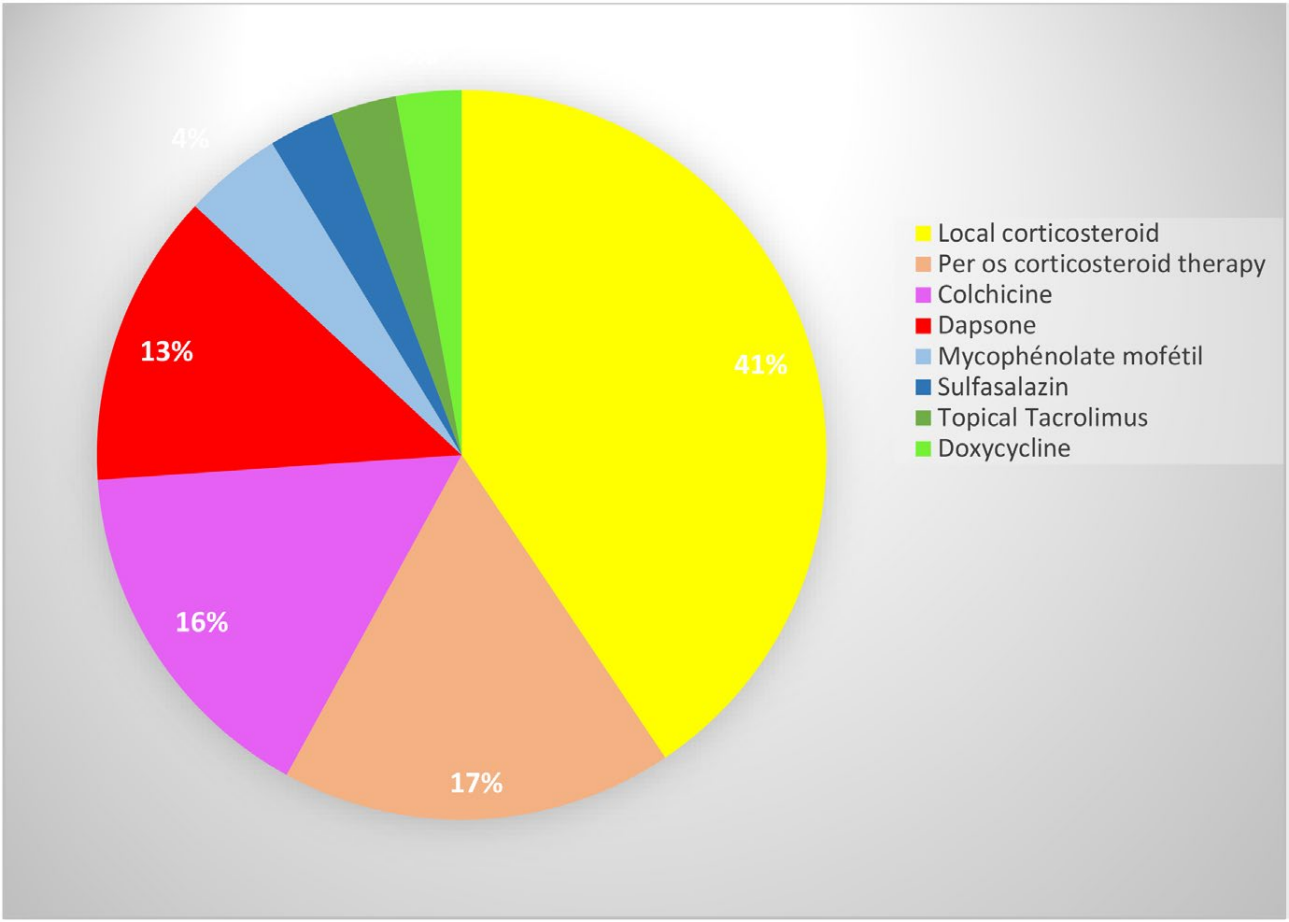
Disappearance of the Nikolsky's sign according to the number of times the molecule was prescribed, Bordeaux, France, 2022.

**TABLE 1:** Increase in the disappearance of the pinch sign based on the number of times the molecule has been prescribed, Bordeaux (France), 2022.

Treatment	Posology	Time of treatment	Number of prescriptions	Disappearance of the Nikolsky's sign	Missing data
Monotherapy					
Colchicine	0.5–1.5 mg/d	3–12 m	8	5/6 (83, 3)	2
Local corticosteroid	3/d	3–12 m	28	12/23 (52, 2)	5
Dapsone	50–100 mg/d	1–3 m	6	1/6 (16, 7)	0
Per os corticosteroid therapy	1 mg/kg/d^1^	10 d–1 m	7	0/7 (0, 0)	0
Sulfasalazin	0.5 mg/kg/d	15 d	2	0/2 (0, 0)	0
Topical tacrolimus	3/d	1–3 m	1	0/1 (0, 0)	0
Doxycycline	200 mg/d	15 d	1	No applicable	1
Mycophenolate mofetil	1–3 g/d	1 m	1	No applicable	1
Associations					
Dapsone + Per os corticosteroid therapy			3	1/3 (33, 3)	0
Colchicine + Per os corticosteroid therapy			1	1/1 (100, 0)	0
Colchicine + Topical Tacrolimus			1	1/1 (100, 0)	0
Colchicine + Doxycycline			1	No applicable	1
Per os corticosteroid therapy + Mycophenolate mofetil			1	0/1 (0, 0)	0

Abbreviations: d, day; m, month.

^1^ Short course of 1 mg/kg/d for 1 month, followed by a stepwise decrease of around 2.5 mg to reach 7.5–10 mg/d.

This study shows a significant remission of MMP lesions, particularly with local corticosteroids, dapsone in combination with oral corticosteroids and colchicine. Concerning the use of colchicine as a treatment for MMP in the literature, one study from 2011, was listed and published (Chaidemenos et al. 2011). Colchicine proved effective in all 8 out of 12 patients (66.7%), achieving a remission close to that what we achieved in our study (83.3%) and with the same posology. To try to explain the potential of this novating treatment, a recent meta‐analysis suggested that colchicine disrupts several inflammatory pathways and modulates innate immunity (Leung, Yao Hui, and Kraus 2015). It also reduces the production of certain inflammatory mediators such as leukotrienes and interleukin‐1, as well as certain molecules responsible for chemotaxis by immune cells, notably neutrophils. The anti‐inflammatory action of colchicine with its involvement in innate immunity could explain its mechanism of action. Concerning the other treatments, in our study, there was a higher frequency of adverse events with dapsone such as in cases reported in the literature. In a randomized controlled trial, 19 out of 20 patients developed hemolysis, leading to discontinuation of treatment (Hegarty et al. 2010). Our study contributes to reinforce this starting literature. The low prevalence of the disease justifies the small sample size of our study. Another strength is that follow‐up was carried out by a single, experienced practitioner, which could have reduced potential assessment bias, often criticized in retrospective studies. This study presents some limits. The design of this retrospective exploratory study and the size of the sample did not allow to draw definitive conclusion about efficacy of colchicine to treat oral MMP. In addition, it presented numerous other biases. One of them is that local corticosteroids were systematically associated with the other second‐line treatments not allowing to describe the evolution of the second line of treatment alone. Also, oral hygiene, considered as factor influencing local inflammation of the oral cavity, was not recorded. Other confounding biases inherent to this type of study should be noted: the treatment durations and evaluation periods were not comparable across all the drugs, nor were the prescribing baselines. However, if we refer to dapsone, it was generally prescribed as a second‐line treatment between 2012 and 2016, whereas colchicine was prescribed between 2016 and 2021. Further study, with higher level of evidence, needs to be conducted, taking into account such factors would also gain to report the exact posology and not only the molecules prescribed, and also evaluation of the pain with a graduate scale. A multicenter study with more patients would enable to perform subgroup analyses.

This study highlighted the promising potential of colchicine for the treatment of oral MMP that could be propose as a second‐line of treatment instead of dapsone usually used and usually poorly tolerated. Higher‐level evidence studies would be interesting to confirm these results.

## Author Contributions

**E. Fribourg:** data curation, writing – original draft, investigation. **V. Chuy:** methodology, data curation, formal analysis. **M. Fenelon:** supervision. **S. Catros:** supervision. **J.C. Fricain:** validation, conceptualization, supervision, resources.

## Conflicts of Interest

The authors declare no conflicts of interest.

## Data Availability

The data that support the findings of this study are available from the corresponding author upon reasonable request.
